# Effect of Sulfate Attack on the Expansion Behavior of Cement-Treated Aggregates

**DOI:** 10.3390/ma17030660

**Published:** 2024-01-29

**Authors:** Qi Wang, Jiankun Liu, Pengcheng Wang, Jiangxin Liu, Mingzhi Sun

**Affiliations:** 1 Research Institute of Highway Ministry of Transport, Beijing 100088, China; 2 School of Civil Engineering, Sun Yat-sen University, Guangzhou 510006, China; 3 State Key Laboratory for Tunnel Engineering, Guangzhou 510275, China; 4 Railway Engineering Research Institute, China Academy of Railway Sciences Corp., Ltd., Beijing 100081, China

**Keywords:** sulfate attack, cement-treated aggregates, expansion behavior, Sobol sensitivity analysis, dominant factor

## Abstract

The expansion induced by sulfate attack on cement-treated aggregates (SACA) is a well-known problem that can be solved. It causes obvious heaves in road bases and railway subgrades. In this paper, the effects of the sodium sulfate content, cement content, degree of compaction, sulfate types, attack types, aluminum ion supply, concentration of curing sulfate solution, and temperature on the expansion behavior induced by SACA were investigated over 60 days in the laboratory. Based on the Sobol sensitivity analysis method, the influence of the sensitivity of each factor on the expansion was quantitatively analyzed, and the dominant factor of expansion was proposed. Results show that sulfate content is the domain factor of expansion that is induced by SACA, and it presents a logarithmic function relationship with strain. The 0.5% sodium sulfate content is the minimum sulfate content which causes the expansion that is induced by SACA. When the sulfate content is less than 1%, the expansion induced by SACA is minor. When the sulfate content is between 1% and 3%, the expansion behavior is expressed in four stages as follows: rapid strain increase, followed by a short stagnation period, then a significant strain increase and, finally, constant strain. When the sulfate content is greater than 5%, there are two stages comprising the expansion behavior as follows: the rapid strain increases and constant strain occurs. Greater sulfate content, greater degree of compaction, and lower temperature have positive effects on the expansion induced by SACA. The cement content does not have a consistent effect on expansion behavior. Compared with a sodium sulfate attack, both the reaction rate and expansion of cement-treated aggregates that are attacked by gypsum are smaller, and the attack period is also longer. When the sulfate content is greater than 1%, the addition of kaolin promotes the progression of the expansion induced by SACA. A small amount of water is sufficient for the demand for the sulfate attack. When the sulfate content is at a certain level, the expansion induced by SACA that is under external attack is much smaller than the expansion that is under internal attack. This study is expected to serve as a reference for future research on the mechanics of SACA, and it attempts to provide theoretical support for amending expansions that are induced by SACA.

## 1. Introduction

Cement-treated aggregates have been widely used in modern construction, such as railway subgrades and road bases. However, when the surrounding environment or the soils contain sulfate, it may cause damage to the cement-treated aggregates, such as expansion, cracking, loosening, and materials peeling off [1,2,3]. Sulfate attack on cement-treated aggregates (SACA) is a well-known problem that causes severe damage to concrete structures in addition to remarkable heave. In northwest China, continuous heave was observed in a high-speed railway subgrade section, and cement-treated graded macadam, ettringite (3CaO·Al_2_O_3_·3CaSO_4_·32H_2_O), gypsum (CaSO_4_·2H_2_O), and thaumasite (CaCO_3_·CaSO_4_·CaSiO_3_·15H_2_O) were found in the subgrade bed [4]. In Tarragona, Spain, the embankments were designed by including soil–cement-treated transition wedges (with gypsum), and they experienced a continuous and severe heave shortly after construction. The ettringite and thaumasite in the embankment were analyzed [5]. In northern Louisiana, the roads using Winn rock (CaSO_4_) gravel as a surface course were rehabilitated via stabilization with Type I Portland cement and an asphaltic overlay, and it heaved within days. The ettringite was analyzed in the expansion position [6].

Although discussion regarding the sulfate attack mechanism has been ongoing [7,8,9,10], it is readily admitted that the expansive products (e.g., ettringite, gypsum, and thaumasite) involved in a sulfate attack led to the expansion induced by SACA. The general reaction which causes the attack may be summarized in Equations (1)–(3) [11,12]. The formation of crystals in a supersaturated solution may exert pressure on its surroundings and lead to expansion [13].

(1) $${Ca(\mathrm{OH})}_{2}+{\mathrm{SO}}_{4}^{2-}+2H_{2}O\to {\mathrm{CaSO}}_{4}\cdot 2H_{2}O\left(\mathrm{gypsum}\right)+{2\mathrm{OH}}^{-}$$

(2) $$6{\mathrm{Ca}}^{2+}+2\mathrm{Al}{\left(\mathrm{OH}\right)}_{4}^{-}+{4\mathrm{OH}}^{-}+3{\left({\mathrm{SO}}_{4}\right)}^{2-}+26H_{2}O\;\to 3\mathrm{CaO}\cdot {\mathrm{Al}}_{2}O_{3}\cdot 3{\mathrm{CaSO}}_{4}\cdot 32H_{2}O\;(\mathrm{ettringite})$$

(3) $$3\mathrm{CaO}\cdot 2\mathrm{Si}O_{2}\cdot 3H_{2}O+{2\{\mathrm{CaSO}}_{2}\cdot 2H_{2}O\}+{\mathrm{CaCO}}_{3}+{\mathrm{CO}}_{2}+32H_{2}O\;\to {\mathrm{CaCO}}_{3}\cdot {\mathrm{CaSO}}_{4}\cdot {\mathrm{CaSiO}}_{3}\cdot 15H_{2}O\;(\mathrm{thaumasite})$$

The conditions of expansion induced by SACA have been studied by many researchers. The cations (i.e., Na^+^, K^+^, Mg^2+^, Ca^2+^) also have a major influence on the reaction’s development [14]. Gypsum and M-S-H are the major products when Mg^2+^ is present in the sulfate solution, whereas ettringite and gypsum are predominantly found in the presence of Na^+^ [15]. Water is the reactant for the formation of ettringite, which is a prerequisite for SACA [16]. In an alkaline environment (pH > 10.5), aluminate and silicate ions from the clay minerals of soil are released (in potentially large quantities), and then they are made available for the SACA reaction [17]. Ma et al. [18] proposed that when mortar attacks a 30 g/L sulfate ion solution, it could induce expansion. Puppala et al. [19] concluded that the sulfate content that causes soil expansion is between 320 and 43,500 mg/kg. Wang et al. [6] proposed that the expansion induced by SCAC is proportional to the cement content. Ouyang et al. [20] found that although aggregates had higher initial strength when the cement content was high than when it was low, it expressed greater expansion after the sulfate attack. Temperature also has a strong effect on the SACA reaction, and 0 to 20 °C is the active temperature range for a sulfate attack [4]. Tai et al. [21] noted that porosity could also have a positive effect on durability by generating additional space to accommodate the expansive phase. Through field tests, Tang et al. [22] proposed that the temperature, sodium sulfate, and cement content may be the main causes of arch expansion on pavement surfaces and cement-stabilized macadam bases. These factors may change the period of sulfate-induced distress initiation from 2 to over 25 years [14].

Based on the overview of the literature described above, it is worth noting that although the factors that influence the SACA reaction are known, there is no unified standard for the critical sulfate content of expansion induced by SACA. The cement content and the degree of compaction for expansion behavior induced by SACA have seldom been analyzed. Moreover, the quantitative analysis of the influencing factors on expansion induced by SACA has also been rarely analyzed. Therefore, the objective of this paper lies in studying the effects of the sodium sulfate content, cement content, degree of compaction, sulfate types, attack types, aluminum ion supply, concentration for curing the sulfate solution, temperature for the sulfate attack cement-treated aggregate, and the dominant factor of expansion induced by SACA. Through the swelling tests at the constant environment of 10 °C temperature and 70% humidity, the expansion behavior induced by SACA has been monitored up to 60 days. Based on the Sobol sensitivity analysis method, considering the interactions among influencing factors, the sensitivity of each factor affecting the SACA has been quantitatively analyzed, and the dominant factor of expansion induced by SACA has been analyzed. This work reveals the influence factors of SACA and explores the dominant factor of SACA. This work is expected to provide a solid base of information to understand the expansion mechanism of SACA, and it aims to serve as a reference for the design and construction of high-speed railway lines and maintenance of subgrade heave.

## 2. Materials and Methods

The high-speed railway (HSR) in northwest China was experiencing a continuous and severe heave. The subgrade was approximately 3.2 m high, and the filling of the transition zone was cement-treated graded macadam of different cement content, 5% for the top 0.4 m, and 3% for the remaining part. The surface aggregates of the subgrade bed were treated with 3% and 5% content of ordinary Portland cement. The field heave at different depths from November 2017 to April 2018 was monitored, and it was found that the cement-treated graded macadam filler is the main location of expansion [4]. The surface aggregates of the subgrade bed were treated with 3% and 5% content of ordinary Portland cement. The mineral composition of the subgrade bed has been analyzed, which is mainly composed of quartz, calcite, and albite. The sulfate content on the top of the subgrade was close to 0.3% after expansion. Some products of SACA (e.g., ettringite, thaumasite, and gypsum) have been analyzed in the surface layer of the subgrade bed. 

### 2.1. Materials

To simulate the field conditions in the laboratory, the fresh stone powders (without salt) with a diameter of less than 2 mm were used in this study. The stone powder contained albite (42%), quartz (19%), calcite (14%), microline (8%), amphibole (7%), muscovite (6%), and clinochlore (4%). The optimum moisture content (11.2%) and maximum dry density (2.36 g/cm^3^) of the stone powders were obtained. 

Sulfate is the main reactant in the SACA reaction and participates in the formation of ettringite and thaumisate. Considering that the sulfate content was close to 0.3% after the reaction in the field, thus, the initial sulfate content should be much greater than 0.3%. Sodium sulfate is the only source of SACA in this paper; the sodium sulfate content was mainly set to 3% to ensure that the sulfate ions were sufficient for participating in the actual reaction in this study. In the study of sodium sulfate-influencing factors, to explore the lowest sodium sulfate content causing the expansion induced by SACA, the sodium sulfate content was set at 0.1% to 5%. To study the effect of sulfate solubility, a group of specimens of gypsum (with content of 1% to 3.5%) as the medium soluble sulfate as the sulfate source were researched. To study the effect of sodium sulfate supply, the sodium sulfate solution (0.5% to 5% content) curing environment was set. To simulate the external attack, four specimens without sulfate were cured in the sulfate solution as well. The sodium sulfate and gypsum used in this study were reagent grade with a purity of at least 99%.

Ordinary Portland cement 42.5, obtained from Zhucheng Yangchun Cement Co., Ltd., Zhucheng, China, was used in the tests. Considering the cement contents were 3% and 5% of the surface bed and transition zone in the field, the cement content was mainly set to 3% to remain consistent with the condition of the surface bed. In the study of the cement influenced, the cement content was set at 1%, 3%, 5%, and 7%.

To study the influence of aluminum ions, a set of specimens with 4% content kaolin was tested. The degree of compaction affects the swelling potential of the aggregates; thus, a set of specimens with the degree of compaction (0.85 to 0.97) was studied in this paper. 

Considering 0 to 20 °C is the active temperature range for sulfate attack reactions [4], the curing temperature of 10 °C was chosen in this paper. Considering that water, sulfate, and temperature play important roles in sulfate attack, thus, the deionized water and temperature (20 °C) curing environment was set to study the influence of water and temperature.

The specimen preparation matrix and curing conditions are shown in Table 1, Table 2, Table 3, Table 4, Table 5 and Table 6.

### 2.2. Experimental Procedures

**Figure 1:**
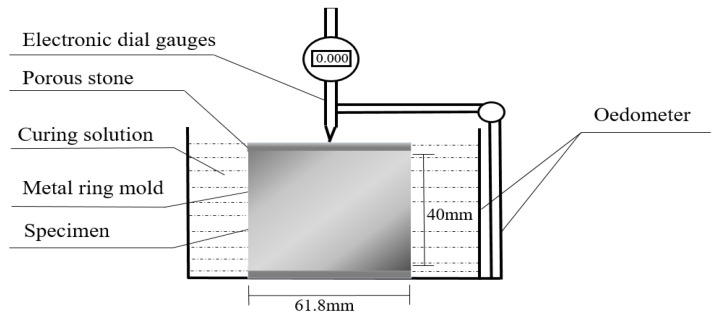
Details for swelling tests device.

The amount of stone powder, cement, sodium sulfate, and deionized water was calculated based on the maximum dry density, the optimum moisture content of fresh basalt powders, and the degree of compaction (Table 1, Table 2, Table 3, Table 4, Table 5 and Table 6). The process of the materials mixing refers to the Code for Soil Test of Railway Engineering [23]. The stone powders were mixed with sodium sulfate. Then, the deionized water was added to the mixture and stirred until homogeneous. Next, the cement was added to the mixture and homogeneously stirred. Then, the material above was compacted into a metal ring mold with 61.8 mm diameter and 40 mm height. After compaction, porous stones were placed on two sides of the specimen and transferred to an oedometer. The electronic dial gauge was fixed by the oedometer to measure the vertical swelling (Figure 1). 

At last, the specimens with electronic dial gauges were maintained in a test chamber with a certain temperature and humidity. The initial value of the electronic dial gauge was set to zero, and the expansion behavior of SACA was monitored up to 60 or 240 days. In this study, the strain of the specimen was calculated by the value of vertical swelling (∆H) and the initial height of the specimen ($H_{0}$) (Equation (4)).

(4) $$\varepsilon =\frac{∆H}{H_{0}}\times 100\%$$

## 3. Results and Discussion

### 3.1. Sodium Sulfate Content

**Figure 2:**
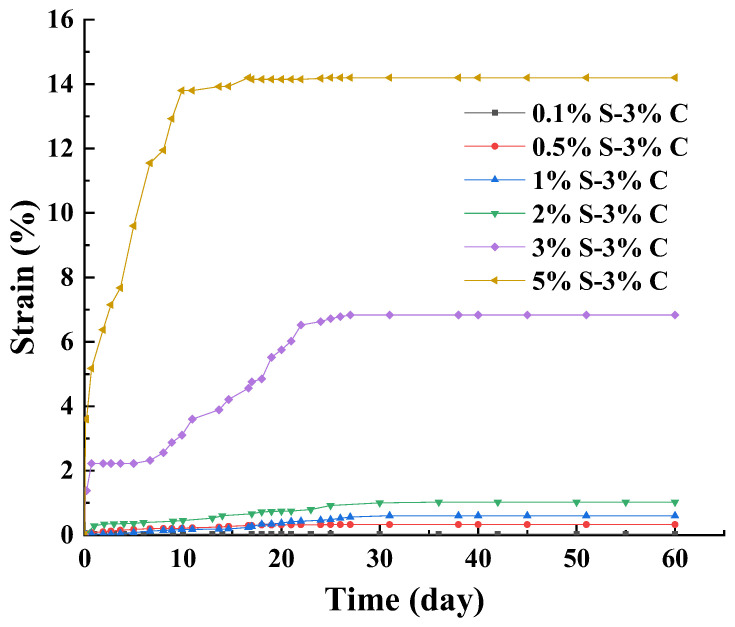
Strain curves of sulfate attack over time with different sodium sulfate content.

Figure 2 illustrates the strains increased with the increase in sodium sulfate content. The $\varepsilon_{d60}$ represents the strains of specimens at day 60. From Figure 2, it can be seen that the 0.1% S–3% C did not experience the expansion under SACA. The $\varepsilon_{d60}$ of 0.5% S–3% C, 1% S–3% C, 2% S–3% C, 3% S–3% C, and 5% S–3% C were 0.33%, 0.6%, 1.0%, 6.8%, and 14.2%, respectively. Thus, the 0.5% sodium sulfate content is the minimum sulfate content causing the expansion induced by SACA. The strain of 5% S–3% C was the largest, which increased rapidly in the first 20 days and then reached a stable stage. The strains of 0.5% S–3% C, 1% S–3% C were small, and the strain curves can be divided into two stages: it increased rapidly in the first 30 days and then reached a stable stage. The strain curves of 3% S–3% C can be generally divided into four stages: the specimens expanded rapidly in the first 2 days; then, there was a short stagnation period from day 2 to day 5; there was a continuous heave from day 5 to day 25 and a constant strain from day 25 to day 60.

Sodium sulfate plays an important role in the formation of expansive products (i.e., ettringite and gypsum). The formation of gypsum could lead to a crystallization pressure of not more than 3 MPa [24]. The maximum expansion pressure resulting from ettringite formation in OPC based mortars can reach about 8 MPa, exceeding the tensile strength of the hardened cement paste matrix leading to crack formation and structural deterioration of the binder matrix [25].

When the sodium sulfate content is less than 1%, the attack products are mainly composed of ettringite, followed by gypsum, and mirabilite is hardly produced. Thereafter, with the increasing of sulfate content, the mirabilite is formed and became the major product gradually [26]. Under the 1% sulfate content, the lower expansion rate during the early stage was due to the filling up of concrete pores by these products. But a further accumulation of ettringite and gypsum caused the concrete to expand at the later stage [27]. The increasing sulfate content was more conducive to the formation of mirabilite. When sulfate content was larger than 3%, the specimens expanded greatly. With the sodium sulfate content increasing, the morphology and structure of cementitious materials are increasingly rough due to the columnar mirabilite crystallization and pores increasing [22]. Thus, under the 3% and 5% sulfate content, the formation of mirabilite led to the strain increasing obviously.

### 3.2. Cement Content

**Figure 3:**
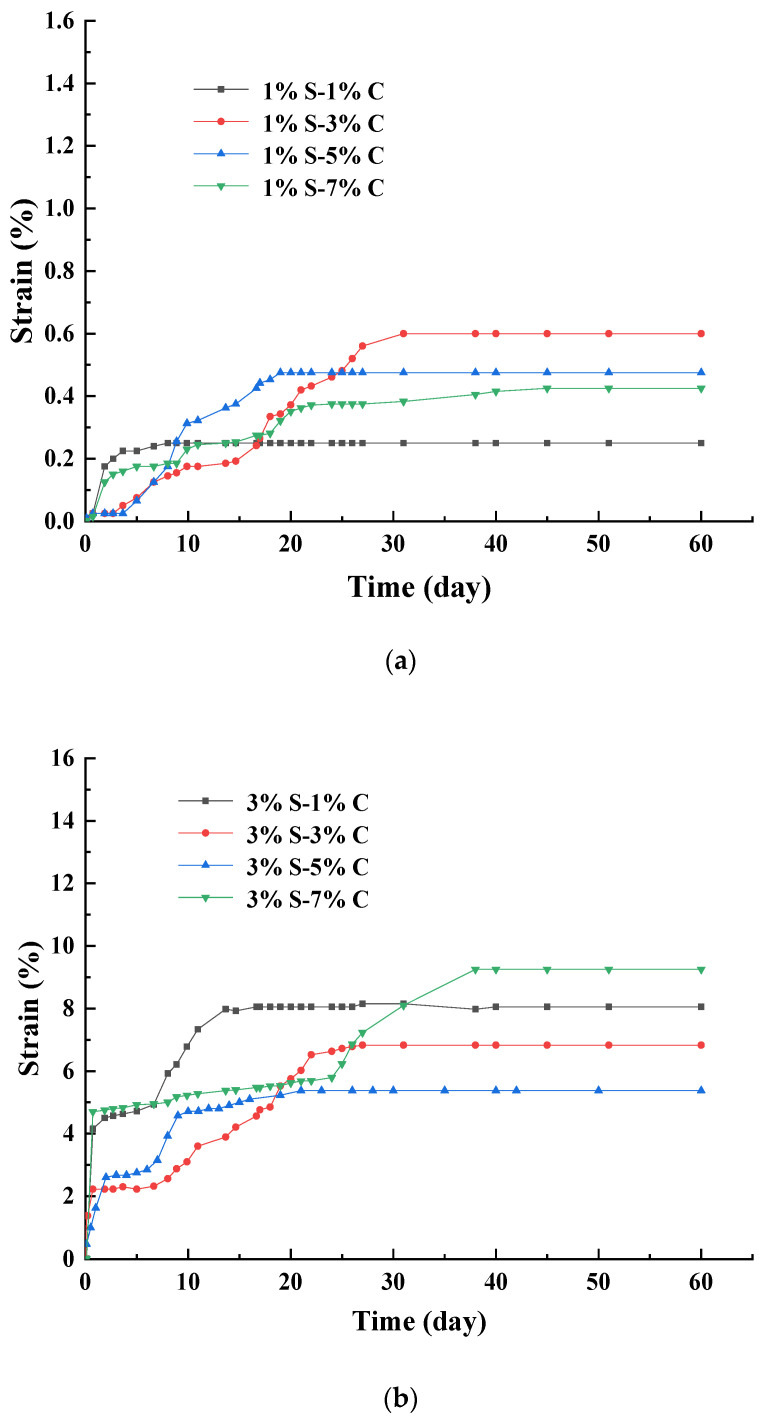

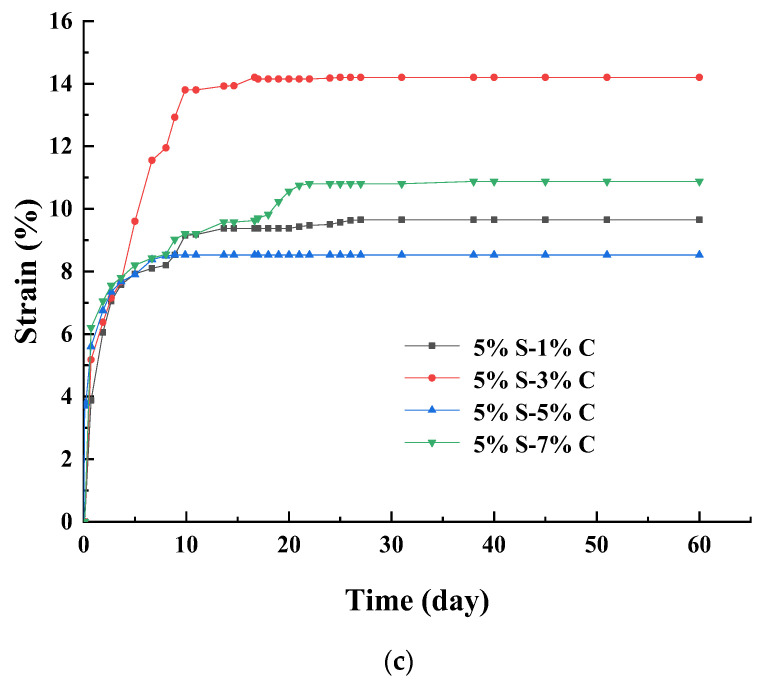
Strain curves of sulfate attack with different cement content. (**a**) Strain curves of sulfate attack with content under 1% sodium sulfate content. (**b**) Strain curves of sulfate attack with content under 3% sodium sulfate content. (**c**) Strain curves of sulfate attack with content under 5% sodium sulfate content.

The influence of cement content on SACA can be seen in Figure 3. Under different sodium sulfate contents (1%, 3%, and 5%) and cement contents (1%, 3%, 5%, and 7%), the strains first increased and then reached a stable stage with the increasing of curing time within 60 days. From Figure 3a–c, it can be seen that at a certain sodium sulfate content, $\varepsilon_{d60}$ was not increased with the increasing of cement content (1%, 3%, 5%, and 7%). At 1% sodium sulfate content, the $\varepsilon_{d60}$ increased at first and then decreased (0.25%, 0.60%, 0.48%, and 0.43%) with the increasing of cement content. Under 3% sodium sulfate content, the $\varepsilon_{d60}$ decreased at first and then increased (8.05%, 6.83%, 5.38%, and 9.25%) with the increasing of cement content. At 5% sodium sulfate content, $\varepsilon_{d60}$ showed a fluctuating trend (9.65%, 14.2%, 8.53%, and 10.88%) with the increasing of cement content. Thus, at a certain sodium sulfate content, $\varepsilon_{d60}$ was not increased with the increasing of cement content. Most samples did not reach the maximum or minimum value at the 7% or 1% cement content. It also can be seen that under the different cement content, the strains of specimens with 5% sodium sulfate were greater than those with 3% sodium sulfate, and it was larger than those with 1% sodium sulfate. This means the sulfate content expressed a stronger effect on strain than cement content.

In sulfate attack, the cement hydration products (e.g., calcium silicate hydrate, calcium hydroxide) are the main reactants of the sulfate attack reaction, which participates in the formation of expansive minerals [28]. The calcium hydroxide leaching can bring into the solution Ca^2+^ ions available for the precipitation of ettringite [7]. However, cement also plays a role for aggregates hardened, and it reduces the permeability and porosity of the filler [20]. Thus, the excess cement may hinder the development of the expansion of aggregate, which results in the cement content not expressing a consistent tendency for expansion behavior.

### 3.3. Aluminum Ions

**Figure 4:**
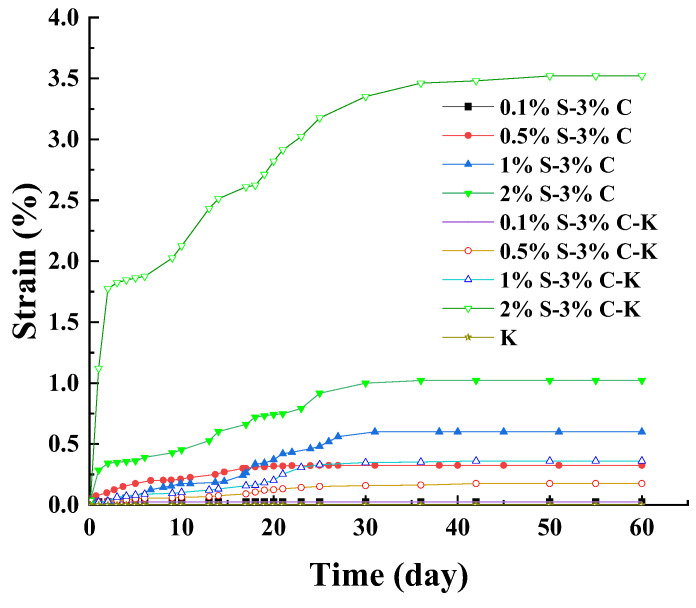
The effect of kaolin on the strain of sulfate attack.

It can be seen from Figure 4 that the specimen of K did not experience expansion. This means that the kaolin itself could not induce the expansion of aggregates. The $\varepsilon_{d60}$ of 0.5% S–3% C–K, 1% S–3% C–K, and 2% S–3% C–K reached 1.8%, 3.6%, and 3.5%, respectively. Compared with 0.5% S–3% C, 1% S–3% C, and 2% S–3% C ($\varepsilon_{d60}$ reached 0.6%, 1.0%, and 1.02%), the strain of 0.5% S–3% C–K, 1% S–3% C–K, and 2% S–3% C–K increased significantly. The high-C_3_A mortars suffered significantly greater expansion than the low-C_3_A mortars under sulfate attack [29]. This is because the aluminum ions play an important role in the formation of ettringite. The kaolin contains a large amount of aluminum ions, which participate in the formation of ettringite and promote the reaction of sulfate attack. This means the addition of kaolin promotes the sulfate attack reaction and the expansion developed. At 0.1% sodium sulfate content, the specimen of 0.1% S–3% C–K is still not swelling. The sulfate attack hardly occurred at 0.1% sulfate content; thus, the addition of kaolin could not promote the development of expansion.

### 3.4. Medium Soluble Sulfate

**Figure 5:**
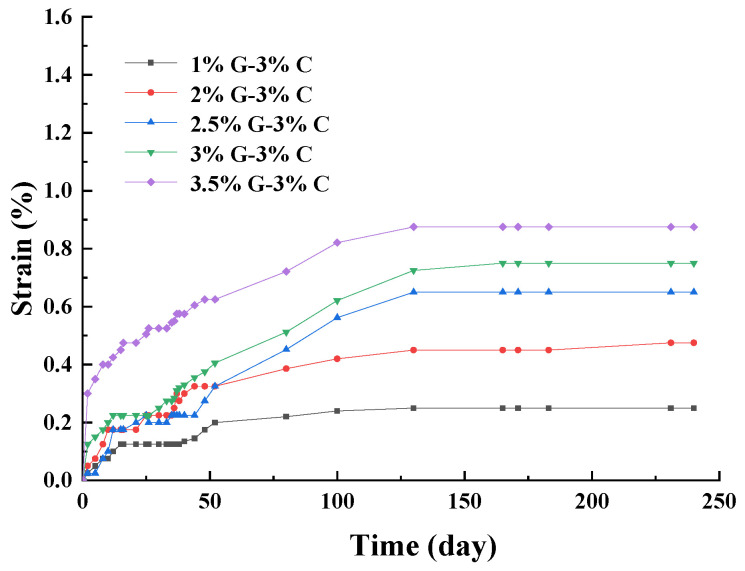
Strain curves of sulfate attack over time with different gypsum contents.

It can be seen in Figure 5 that the strains of the specimens increased with the increasing of gypsum content. The $\varepsilon_{d240}$ represents the strains of specimens at day 240. The strain of 1% G–3% C was the smallest, and the $\varepsilon_{d240}$ was about 0.25%. The strain of 3.5% G–3% C was the largest, and the $\varepsilon_{d240}$ was about 0.88%. The $\varepsilon_{d240}$ of 2% G–3% C, 2% G–3% C, and 2.5% G–3% C reached 0.48%, 0.65% and 0.75%, respectively. The five specimens exhibited the same strain tendencies on the whole. The strains of specimens grew rapidly in the first 30 days of the reaction, and the strains of some specimens grew slowly in 30 to 50 days. Then, the strains also increased continuously in 50 to 150 days. The strain reached a stable stage in 200 to 240 days. However, the strain of specimens under sodium sulfate attack reached a stable stage for about 30 days (Figure 2). Gypsum is a medium-soluble salt, its dissolution rate is slow and the ionization rate is much smaller than that of sodium sulfate, which results in a longer attack time than that of sodium sulfate. This also leads to the strains of the samples attacked by gypsum at day 240 being still less than those attacked by sodium sulfate at day 60.

### 3.5. Degree of Compaction

**Figure 6:**
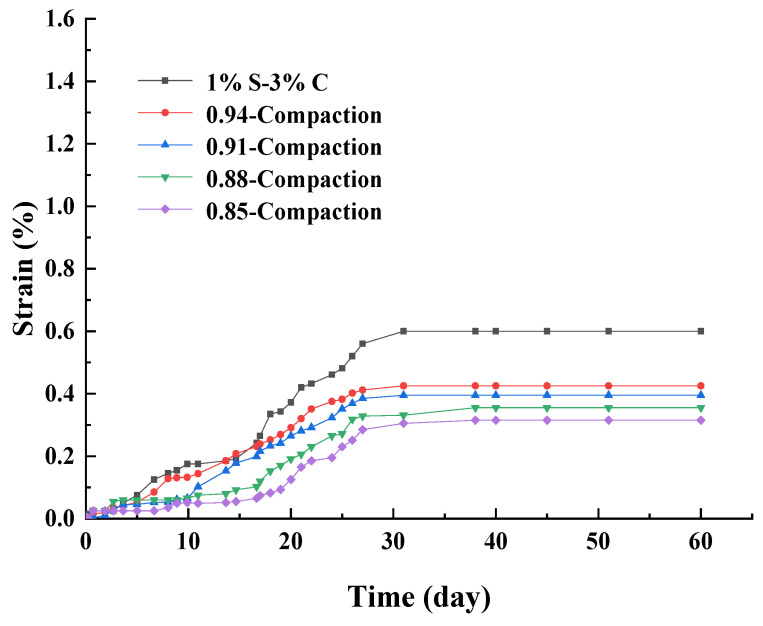
Strain curves of sulfate attack over time with different compaction degrees.

Figure 6 shows that under 1% sulfate content and 3% cement content, the strain curves of specimens have the degrees of compaction of 0.85, 0.88, 0.91, 0.94, and 0.97. All of the five specimens exhibited the same strain tendencies. On the whole, the strain decreased with the decreasing of the degree of compaction. The degree of compaction of 1% S–3% C was 0.97, which was the largest strain ($\varepsilon_{d60}$ reached about 0.6%). And the strain of the 0.85 compaction was the smallest ($\varepsilon_{d60}$ reached about 0.32%). The $\varepsilon_{d60}$ values of 0.94 compaction, 0.91 compaction and 0.88 compaction were 0.43%, 0.40% and 0.36%, respectively. 

The pore structure of the soil affects the swelling reaction [16]. The continuous pore system favors the convective transport of sulfate solution within the cement-treated aggregates, and the expansive products formed in the pores. Under SACA, the larger the degree of compaction, the smaller the pore volumes in aggregates, and the tighter the arrangement among aggregates, which results in a higher expansion risk of cement-treated aggregates. Thus, compared with 1% S–3% C, the strain growth rates of specimens with a smaller degree of compaction were slower. This is due to the specimens with the smaller degree of compaction being looser, and more pores need to be filled in the specimen, providing more free space for the accommodation of products [30] and leading to the smaller strain. 

### 3.6. Curing Solution

#### 3.6.1. Deionized Water

**Figure 7:**
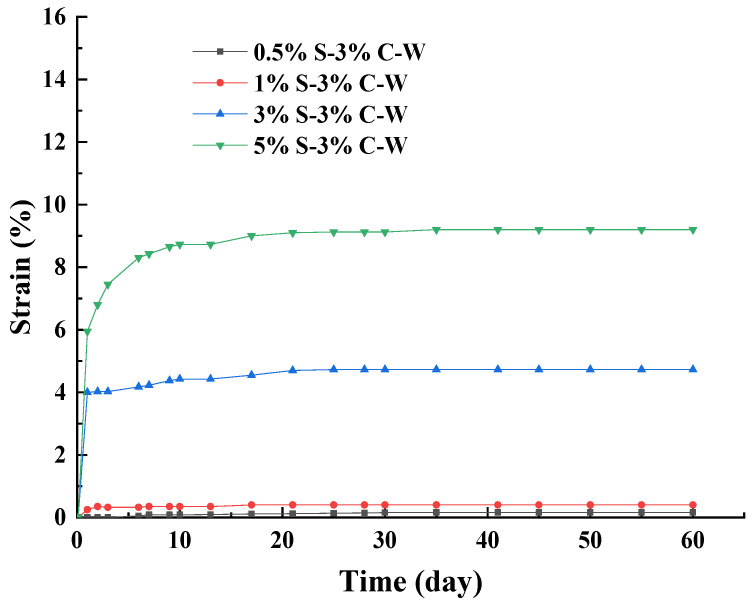
Strain curves of sulfate attack over time under deionized water curing environment.

Figure 7 shows the strain curves of SACA curing in deionized water. The strains increased with the increasing of sulfate content. Under 0.5% and 1% sodium sulfate content, the expansion of specimens was small, and the strain of 0.5% S–3% C–W was the smallest. The strain of 5% S–3% C–W was the largest. The $\varepsilon_{d60}$ of 0.5% S–3% C–W, 1% S–3% C–W, 3% S–3% C–W, and 5% S–3% C–W reached about 0.16%, 0.4%, 4.7% and 9.2%, respectively. Under 3% and 5% sodium sulfate content, the strain increased rapidly in the first 10 days and then reached a stable stage after 30 days.

Compared with Figure 2, it can be seen that under the same sodium sulfate content, the strain of the specimens under the deionized water curing environment was smaller than that under the 70% humidity curing environment. The water in specimens continued volatilization with the curing time increasing under the 70% humidity curing environment, which leads to the solubility of sodium sulfate decreasing and results in the continuous crystallization of expansive products and expansion of aggregates. Under the deionized water curing environment, the sodium sulfate in the specimen was continuously dissolved in the deionized water, thereby diluting the sodium sulfate concentration in the specimen, which results in the reaction rate of sulfate attack decreasing. Therefore, the strain of specimens curing in deionized water was smaller than that in the 70% humidity curing environment.

#### 3.6.2. Sodium Sulfate Solution

(1) Simultaneous attack of internal and external

**Figure 8:**
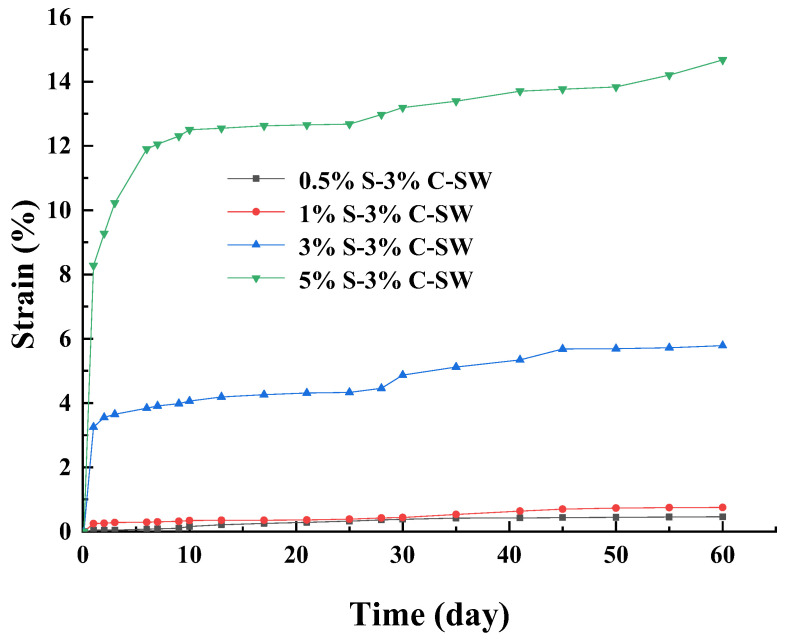
Strain curves of sulfate attack over time under sodium sulfate solution curing environment.

It can be seen in Figure 8 that the strain of the specimens curing in sodium sulfate solution increased with the increasing of sodium sulfate content. The strain increased continuously within 60 days on average. The strain curves of specimens under 3% and 5% simultaneous internal and external attack can be divided into two stages: growth rapidly within 30 days and then increasing slowly after 30 days. The strain curves of specimens under 0.5% and 1% simultaneous internal and external attack grow slowly. The strain of 5% S–3% C–SW was the largest, and the $\varepsilon_{d60}$ was about 14.7%. The strain of 0.5% S–3% C–SW was the smallest, and the $\varepsilon_{d60}$ was about 0.47%. The $\varepsilon_{d60}$ of 1% S–3% C–SW and 3% S–3% C–SW were 0.75% and 5.78%, respectively. Compared with Figure 7, at the same sodium sulfate content, the strain of the specimen cured in the sodium sulfate solution was greater than that cured in deionized water. The specimens cured in sodium sulfate solution were supplemented with sulfate constantly, which promoted the attack reaction continuously.

(2) External attack

**Figure 9:**
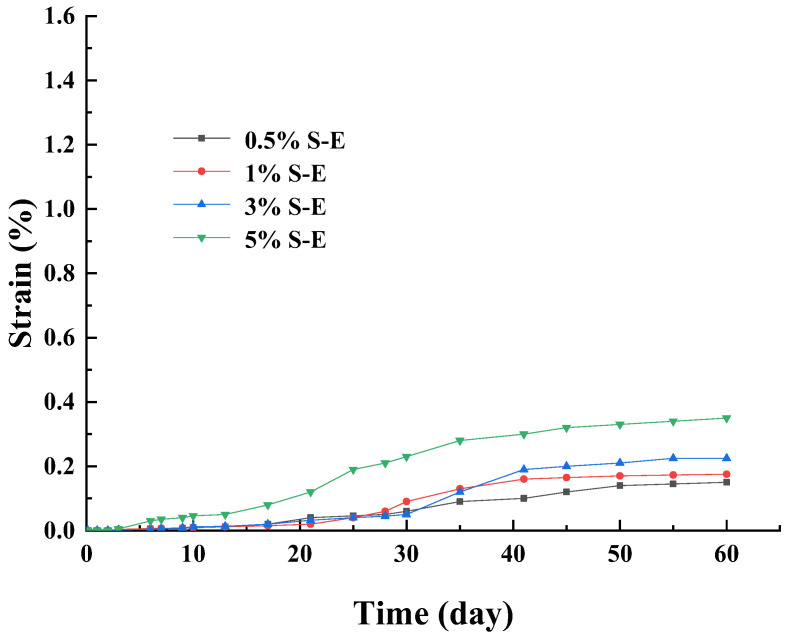
Strain curves of external sulfate attack over time.

Figure 9 shows the strain of SACA under external attack increased continuously within 60 days, and it also increased with the increasing of the concentration of sodium sulfate solution. In addition, at the same sodium sulfate content, the strain of the specimen under external attack was smaller than that under internal attack. The specimens of 0.5% S–E, 1% S–E, 3% S–E, and 5% S–E did not contain sulfate; thus, in the first 3 days, we did not observe expansion. The strain was also small during the first 10 days. As the sulfate attack progressed, sulfate migrated into the specimens and reacted with the hydration products of cement, leading to the formation of ettringite. Thus, the strain increased sustainability after 30 days. The $\varepsilon_{d60}$ of 5% S–E reached 0.35%. On the whole, there is a slight swelling in the cement-treated specimens under external sulfate attack within 60 days. This finding is similar to the research by Wu et al. [31]. He immersed the Portland–limestone cement paste in the 5%wt sodium sulfate solution at 5 °C and found the paste exhibited little expansion within 90 days.

### 3.7. Temperature

**Figure 10:**
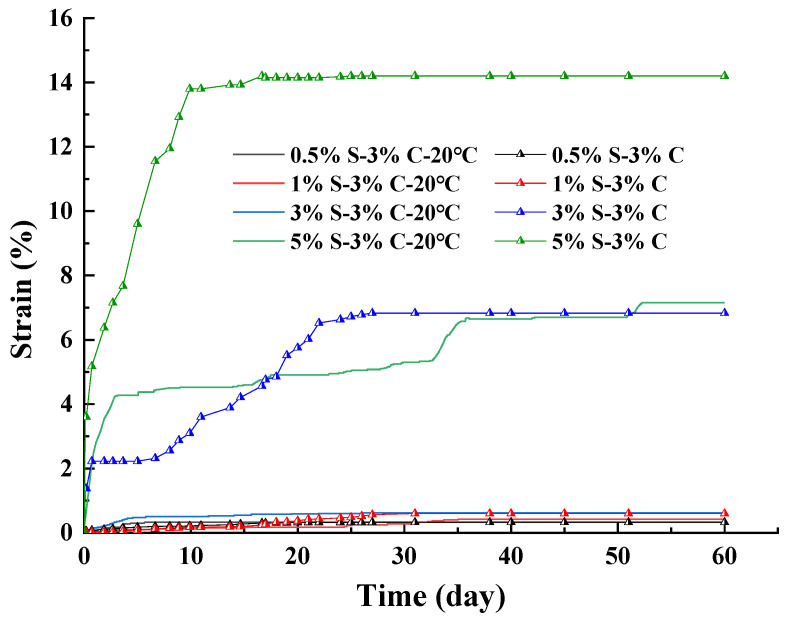
Strain curves of sulfate attack over time at different temperatures.

Figure 10 shows the strain of SACA under curing temperatures of 20 °C and 10 °C. It can be seen that at a certain sodium sulfate content, the strain of specimen curing in 10 °C temperature was greater than that cured at 20 °C temperature. The $\varepsilon_{d60}$ values of 0.5% S–3% C and 0.5% S–3% C–20 °C were about 0.325%. The $\varepsilon_{d60}$ of 1% S–3% C (0.6%) was about 1.4 times that of 1% S–3% C–20 °C (0.425%). The $\varepsilon_{d60}$ of 3% S–3% C (6.83%) was about 10 times that of 3% S–3% C–20 °C (0.625%). The $\varepsilon_{d60}$ of 5% S–3% C (14.2%) was about two times that of 5% S–3% C–20 °C (6.9%).

Comparing with low temperature, the cement hydration rate is faster, and more C-S-H and C-A-H gels are generated at high temperature [32,33]. This means that the sulfate attack reaction should be more intense and experience greater expansion at 20 °C than at 10 °C curing temperature. However, compared with the 20 °C curing temperature, the stain induced by SACA increased 1.4 to 10 times at the 10 °C curing temperature. This is because the solubility of sodium sulfate is reduced at 10 °C, and it is easier to crystallize and precipitate, leading to greater expansion.

### 3.8. Analysis of Dominant Factors

#### 3.8.1. Parameter Setting

Based on the Sobol sensitivity analysis method [34], considering the simultaneous couplings among the influencing factors, the sensitivity of each factor affecting the SACA has been quantitatively analyzed, and the dominant factor of expansion induced by sulfate attack has also been proposed. The influencing factors including sodium sulfate content, cement content, degree of compaction, humidity of curing environment, gypsum content, sodium sulfate concentration of the curing solution, kaolin content, and temperature were designated as X_1_(j), X_2_(j), X_3_(j), X_4_(j), X_5_(j), X_6_(j), X_7_(j), and X_8_(j), respectively. Here, the j = 1, 2, 3 … 46 and 47, which represents the 47 sets of experimental data of SACA in this paper. In the 70% humidity curing environment, the X_4_(j) and X_6_(j) were set to 0.7 and 0, respectively. In the deionized water curing environment, X_4_(j) and X_6_(j) were set to 1 and 0, respectively. In the sodium sulfate solution curing environment, X_4_(j) and X_6_(j) were set to 1 and the corresponding sodium sulfate solution concentration, respectively.

The Sobol sensitivity analysis method considers the total variance of the target is the superposition of the variance generated by a single parameter and the variance generated by the interaction between parameters. The ratio of the variance is regarded as the Sobol sensitivity index. The value of the Sobol sensitivity index indicates the influence of input values on the output values. The larger the value of the Sobol sensitivity index, the more intensity there is between input parameters and output parameters [34].

The Sobol sensitivity analysis of influencing factors was established in Python. Considering the values of influencing factors were not less than 0, thus, the form of the equation was assumed to be Equation (5):

(5) $$\varepsilon_{d60}=a_{1}\ln\left(a_{2}X_{1}+a_{3}\right)\;+b_{1}\ln\left(b_{2}X_{2}+b_{3}\right)+\ldots +h_{1}\ln\left(h_{2}X_{8}+h_{3}\right)$$

#### 3.8.2. Sobol Sensitivity Analysis

**Figure 11:**
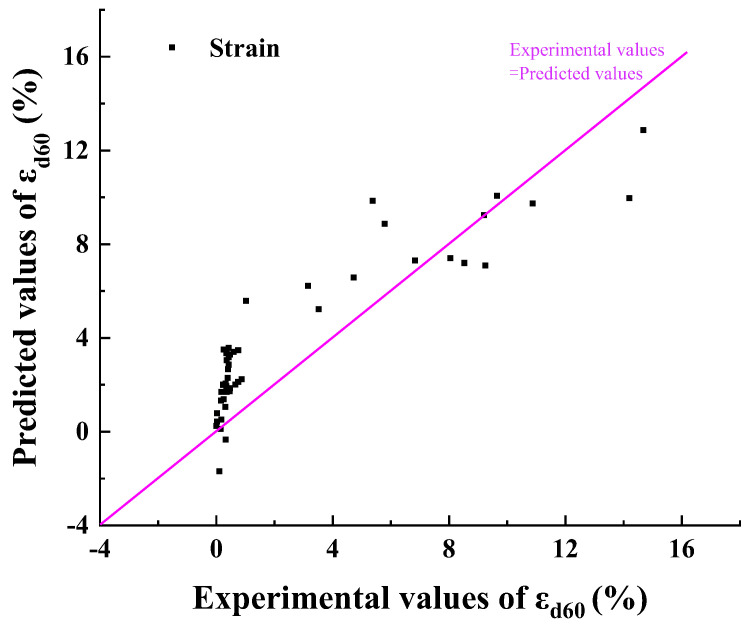
The predicted (based on Equation (5) and Table 7) and the experimental values of ε_d60_.

Table 7 shows the results of each coefficient, and the experimental strain and predicted strain are shown in Figure 11. The goodness of fit (R^2^) was used to reflect the goodness of the fit for the regression model. The value of R^2^ is between 0 and 1, and the closer R^2^ is to 1, the better the fit. Figure 11 shows the comparison between the predicted value and the experimental values of $\varepsilon_{d60}$. The R^2^ was 0.82, which shows a good fitting effect on the whole.

**Figure 12:**
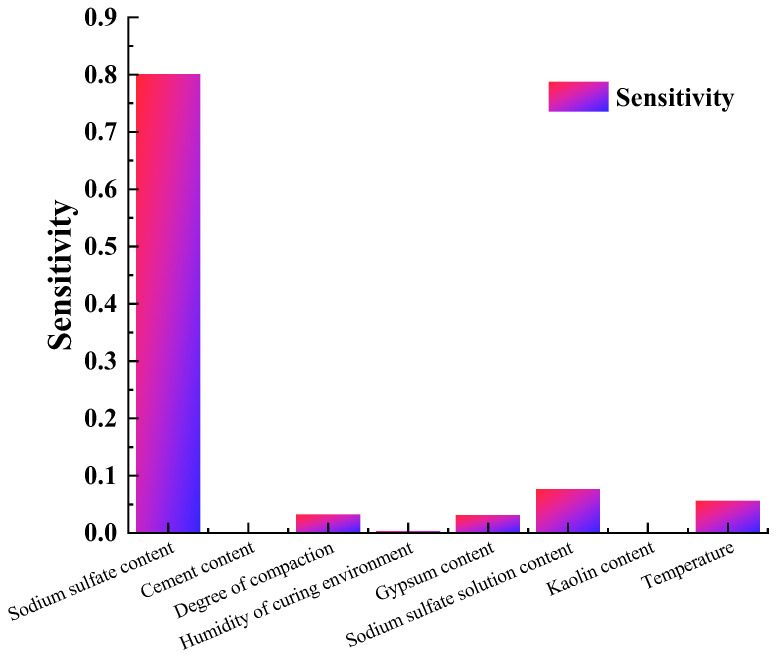
Sensitivities of various factors.

Figure 12 shows the sensitivity analysis results of the influencing factors on strain. It can be seen that the sodium sulfate content exerts an intense effect on the strain, which is followed by the sodium sulfate solution content, temperature, compaction, and gypsum content. The cement content, humidity of the curing environment, and kaolin content show a small impact on the strain. Three of the five factors that largely impact strain were related to sulfate ion content (sodium sulfate content, sodium sulfate solution content, and gypsum content).

**Figure 13:**
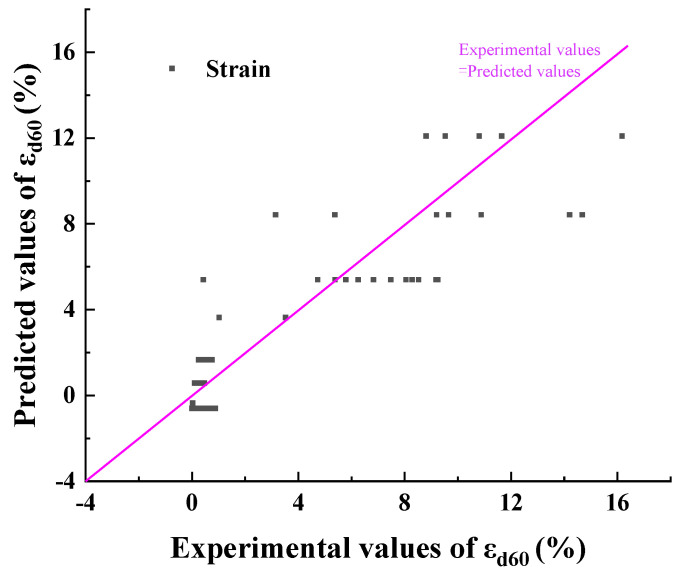
The predicted (based on Equation (6)) and the experimental values of $\varepsilon_{d60}$.

To understand the effect of influencing factors on strain more intuitively, the relationship between sodium sulfate content (X_1_) and strain at day 60 ($\varepsilon_{d60}$) has been analyzed, as shown in Equation (6). The predicted strains based on the relationship between the sodium sulfate content and strain, and the experimental strains are shown in Figure 13. The R^2^ was 0.82, which shows a good fitting effect on the whole.

(6) $$\varepsilon_{d60}=15.99\times \ln\left(0.15X_{1}+0.96\right)$$

In the reaction process of SACA, sulfate ion as the main reactant participates in the formation of expansive products (e.g., gypsum, ettringite, and thaumasite); thus, the sulfate content plays the most important role in the expansion induced by SACA. The sensitivity of sodium sulfate content was significantly greater than that of the sodium sulfate solution content and gypsum content. Comparing with Figure 2 and Figure 5, it can be seen that at the same sulfate content, the strains induced by sodium sulfate were larger than those induced by gypsum. Sodium sulfate is a soluble salt, and gypsum is the medium-soluble sulfate; the dissolution rate of sodium sulfate is faster than that of gypsum. Thus, at the same content of sodium sulfate and gypsum, the concentration of sulfate ions in the sodium sulfate specimen is much greater than that of the gypsum specimen in the first period of reaction. Compared with sodium sulfate attack, the slower ionization rate of sulfate ions in gypsum specimens leads to a longer expansion period under gypsum attack. Compared with Figure 2 and Figure 9, the strains of specimens under internal attack are larger than those under external sulfate attack. Under external sulfate attack, sulfate ions migrate into the specimens at the initial stage of reaction; then, they react with the cement-treated aggregates. Thus, under the same dosage of sodium sulfate, the sulfate concentration of specimens under external sulfate attack is less than that under internal attack, which leads to a smaller strain of specimens under internal attack. 

It can be seen from Figure 12 that the temperature and degree of compaction greatly affect the strain. Here, 0 to 20 °C is the active temperature for expansive products formation. The curing temperature affects the formation rate of expansive product and the solubility of the reactants, thus influencing the expansion of aggregates. The decreasing temperature leads to the solubility decreasing, which results in more precipitation of crystals and larger strain. Under the larger degree of compaction, there are smaller pore volumes in aggregates and a tighter arrangement among aggregates, which results in a greater possibility of expansion with the reaction products generated.

The cement hydration generated the monosulfate hydrated, calcium aluminate hydrated, and calcium hydroxide. The calcium hydroxide and water, monosulfate hydrate, and calcium aluminate hydrate react with sulfate to produce ettringite [35]. The calcium hydroxide reacted with sulfate ions and resulted in the generation of gypsum [35,36]. The gypsum reacted with hydrated calcium aluminate, leading to the formation of monosulfoaluminate hydrate. The monosulfoaluminate hydrate reacted with sulfate ions, resulting in the formation of ettringite [37].

Both the formation of gypsum and ettringite resulted in the expansion of aggregates. Thus, the cement plays an important role in the SACA. However, in Figure 12, the cement shows a small impact on the strain. The solidification of cement reduced the permeability and porosity of the aggregates [20], which prevented the migration of sulfate ions. Thus, the excess cement may hinder the development of the expansion of aggregate. It also can be expressed in Figure 3 that under the different cement content, the strain of the specimen with 5% sodium sulfate was greater than that with 3% sodium sulfate, and the strain of the specimen with 1% sodium sulfate was the smallest. This means that the cement content does not have an obvious impact on the strain, and the sulfate content has a stronger effect on strain than cement content.

The formation of ettringite requires the participation of water molecules, and the sulfate dissolution is also inseparable from water. Moisture plays an important role in sulfate attack. But in Figure 12, the humidity of the curing environment also shows a small impact on the strain, which indicates that a little water was enough for the sulfate attack. In Figure 7, it also can be seen that the specimens are curing in the deionized water with a sufficient supply of water, but the strain was smaller than that of those curing in the 70% moisture environment, which is due to the great quantities of water reducing the concentration.

## 4. Conclusions

In this study, the effects of the sodium sulfate content, cement content, degree of compaction, sulfate types, attack types, aluminum ions supply, concentration of curing sulfate solution, and temperature on the expansion behavior of SACA have been researched. Through swelling tests, the conditions and laws of expansion of SACA have been analyzed. Based on the Sobol sensitivity analysis method, the sensitivity of each factor influencing the SACA has been quantitatively analyzed, and the dominant factor of expansion induced by SACA has also been proposed. The main conclusions can be summarized as follows.

(1) The 0.5% sodium sulfate content is the minimum sulfate content causing the expansion of SACA. Under the sulfate content of less than 1%, the expansion of SACA is small on the whole. Under the sulfate content between 1% and 3%, the expansion behavior expresses four stages: rapid strain increases, a short stagnation period, the strain increasing significantly, and a stable stage with a constant strain. Under the sulfate content larger than 5%, there are two stages of the expansion behavior: rapid strain increase and a constant strain.
(2) The cement content does not express a consistent tendency for expansion behavior induced by SACA. At a certain sulfate content, it takes time for sulfate ions to migrate to aggregates under external attack, which results in the expansion induced by SACA under external attack being much less than that under internal attack. The expansion of SACA under the deionized water curing environment is smaller than that under the 70% humidity curing environment. This is because the concentration of sodium sulfate in the specimen is diluted by deionized water and reduces the rate of the attack reaction. The expansion of SACA at day 60 still expresses a continues increasing under the sodium sulfate solution curing environment.
(3) The expansion induced by SACA under 10 °C curing temperature is obviously larger than that cured in 20 °C curing temperature (the strains increasing about 1.4 to 10 times). The expansion increases with the increasing of the degree of compaction. Under the sulfate content of more than 1%, the addition of kaolin promotes the expansion of SACA. Compared with sodium sulfate attack, when using gypsum as the medium soluble sulfate, the rate of gypsum attack is slower and expansion is smaller.
(4) Based on the Sobol sensitivity analysis method, considering the interactions among influencing factors, it is found that sulfate content is the domain factor of expansion induced by SACA, which also presents a logarithmic function relationship with strain. In addition, the temperature and degree of compaction also have greater influences on expansion.
(5) For cement-treated aggregates in the high-speed railway subgrade, there are no relevant specifications for limiting the sulfate content of sulfate attack. It is recommended to analyze the sulfate ions content in the subgrade filler and the ground soil before subgrade construction. If the sulfate content reaches 0.5%, it is necessary to conduct the swelling testing in the laboratory to simulate the field conditions, judge the swelling potential of the filler and soil, and determine the availability of the filler and soil.

## Figures and Tables

**Table 1:** The effects of cement content under sulfate attack.

Sodium Sulfate Content (%)	Cement Content (%)	Degree of Compaction	Curing Temperature (°C)	Curing Humidity	Designation
1	1	0.97	10	70% humidity	1% S–1% C
	3				1% S–3% C
	5				1% S–5% C
	7				1% S–7% C
3	1				3% S–1% C
	3				3% S–3% C
	5				3% S–5% C
	7				3% S–7% C
5	1				5% S–1% C
	3				5% S–3% C
	5				5% S–5% C
	7				5% S–7% C

**Table 2:** The effects of sodium sulfate content and curing humidity or solution under sulfate attack.

Sodium Sulfate Content (%)	Cement Content (%)	Degree of Compaction	Curing Temperature (°C)	Curing Humidity or Curing Solution	Designation
0.1	3	0.97	10	70% humidity	0.1% S–3% C
0.5					0.5% S–3% C
1					1% S–3% C
2					2% S–3% C
3					3% S–3% C
5					5% S–3% C
0.5				Deionized water	0.5% S–3% C–W
1					1% S–3% C–W
3					3% S–3% C–W
5					5% S–3% C–W
0.5				0.5% sodium sulfate solution	0.5% S–3% C–SW
1				1% sodium sulfate solution	1% S–3% C–SW
3				3% sodium sulfate solution	3% S–3% C–SW
5				5% sodium sulfate solution	5% S–3% C–SW
0				0.5% sodium sulfate solution	0.5% S–E
				1% sodium sulfate solution	1% S–E
				3% sodium sulfate solution	3% S–E
				5% sodium sulfate solution	5% S–E

**Table 3:** The effects of degree of compaction under sulfate attack.

Sodium Sulfate Content (%)	Cement Content (%)	Degree of Compaction	Curing Temperature (°C)	Curing Humidity	Designation
1	3	0.97	10	70% humidity	1% S–3% C
		0.94			0.94–Compaction
		0.91			0.91–Compaction
		0.88			0.88–Compaction
		0.85			0.85–Compaction

**Table 4:** The effects of kaolin under sulfate attack.

Sodium Sulfate Content (%)	Cement Content (%)	Kaolin Content (%)	Degree of Compaction	Curing Temperature (°C)	Curing Humidity	Designation
0.1	3	0	0.97	10	70% humidity	0.1% S–3% C
0.5						0.5% S–3% C
1						1% S–3% C
2						2% S–3% C
0.1		4				0.1%–K
0.5						0.5%–K
1						1%–K
2						2%–K
0	0					K

**Table 5:** The effects of gypsum content under sulfate attack.

Sodium Sulfate Content (%)	Cement Content (%)	Degree of Compaction	Curing Temperature (°C)	Curing Humidity	Designation
1	3	0.97	20	Deionized water	1% G–3% C
2					2% G–3% C
2.5					2.5% G–3% C
3					3% G–3% C
3.5					3.5% G–3% C

**Table 6:** The effects of temperature under sulfate attack.

Sodium Sulfate Content (%)	Cement Content (%)	Degree of Compaction	Curing Temperature (°C)	Curing Humidity	Designation
0.5	3	0.97	20	70% humidity	0.5% S–3% C–20 °C
1					1% S–3% C–20 °C
3					3% S–3% C–20 °C
5					5% S–3% C–20 °C
0.5			10		0.5% S–3% C
1					1% S–3% C
3					3% S–3% C
5					5% S–3% C

**Table 7:** Calculation results of parameters.

Coefficient	Value	Coefficient	Value	Coefficient	Value
$a_{1}$	8.371	$a_{2}$	0.427	$a_{3}$	0.998
$b_{1}$	1.931	$b_{2}$	−0.092	$b_{3}$	3.64
$c_{1}$	10.452	$c_{2}$	1.463	$c_{3}$	−0.545
$d_{1}$	−2.340	$d_{2}$	0.823	$d_{3}$	0.103
$e_{1}$	0.673	$e_{2}$	9.053	$e_{3}$	0.003
$f_{1}$	14.825	$f_{2}$	0.057	$f_{3}$	1.034
$g_{1}$	0.528	$g_{2}$	−0.110	$g_{3}$	0.898
$h_{1}$	1.111	$h_{2}$	−0.644	$h_{3}$	13.114

The coefficients in the list refer to the coefficients in Equation (5).

## Data Availability

Data are contained within the article.
